# *Clostridioides difficile*: A diagnostic intervention

**DOI:** 10.1017/ash.2022.106

**Published:** 2022-05-16

**Authors:** Majd Alsoubani, Joshua Khuvis, Angie Rodday, Shira Doron

## Abstract

**Background:** *Clostridioides difficile* infection (CDI) is a leading cause of healthcare-associated infection and is associated with increased morbidity and mortality. Multiple strategies have been implemented to optimize the diagnostic accuracy of CDI testing algorithms. However, overdiagnosis of *C. difficile* colonization remains a challenge especially in the era of highly sensitive Nucleic acid amplification testing (NAAT). We implemented 2 interventions to reduce the rates of inappropriate *C. difficile* orders and tests. **Methods:** We performed a quasi-experimental retrospective study to examine the rates of all inpatient *C. difficile* test orders and results relative to 2 interventions between January 2018 and February 2021. We defined 3 periods: preintervention, after the first intervention, and after the second intervention. The first intervention, implemented May 2019, was a clinical decision support system (CDSS) tool to guide clinicians to order testing only if CDI criteria were met. The second intervention, implemented July 2020, was the requirement of mandatory antimicrobial team approval of PCR reflex testing for indeterminate toxin or antigen test results. This intervention included a discussion between clinicians and members of stewardship team prior to approval. To evaluate the impact of interventions on ordering appropriateness, chart review was conducted on a random subsample of 100 orders from each period. Hospital-onset CDI (HO-CDI) was calculated using CDC criteria. **Results:** In total, 3,004 *C. difficile* test orders were placed during the study period. The rates of reportable HO-CDI were significantly reduced by 57.1% (*P* = .003). We detected a significant reduction in the number of tests ordered over time from 11.6 to 7.51 per 1,000 patient days. (p **Conclusions:** CDSS tools target patients at high pretest probability of CDI. The restriction of PCR-reflex testing to the antimicrobial stewardship team is a novel effective measure to minimize the misdiagnosis of CDI. The incorporation of multiple strategies is necessary to improve the diagnostic accuracy of *C. difficile* testing.

**Funding:** None

**Disclosures:** None

